# Ultrafast terahertz transparency boosting in graphene meta-cavities

**DOI:** 10.1515/nanoph-2022-0511

**Published:** 2022-11-16

**Authors:** Lan Wang, Ning An, Sen Gong, Xuan Sheng, Yiwei Li, Baicheng Yao, Cui Yu, Zezhao He, Qingbin Liu, Zhihong Feng, Taiichi Otsuji, Yaxin Zhang

**Affiliations:** Yangtze Delta Region Institute (Huzhou), University of Electronic Science and Technology of China, Huzhou, China; Sichuan Terahertz Communication Technology Engineering Research Center, University of Electronic Science and Technology of China, Chengdu, China; Key Laboratory of Optical Fiber Sensing and Communications (Education Ministry of China), University of Electronic Science and Technology of China, Chengdu, China; National Key Laboratory of Application Specific Integrated Circuit, Hebei Semiconductor Research Institute, Shijiazhuang, China; Research Institute of Electrical Communication, Tohoku University, Sendai, Japan

**Keywords:** graphene, terahertz metasurface, ultrafast optics

## Abstract

As an exceptional nonlinear material, graphene offers versatile appealing properties, such as electro-optic tunability and high electromagnetic field confinement in the terahertz regime, spurring advance in ultrashort pulse formation, photodetectors and plasmonic emission. However, limited by atomic thickness, weak light–matter interaction still limits the development of integrated optical devices based on graphene. Here, an exquisitely designed meta-cavities combined with patterned graphene is used to overcome this challenge and promote THz-graphene interaction via terahertz location oscillation. By using an 800 nm pump laser, the local field-induced strong interaction allows sensitive responses to the ultrafast energy transfer from the ultrafast optical pump to graphene electron heat, enabling 46.2% enhancement of terahertz transparency. Such optical modulation of terahertz waves shows ultrafast response in delay less than 10 ps. Moreover, thanks to the nature of graphene, the device shows unique potential for electrically dynamic tuning and further bandwidth broadening.

## 1 Introduction

Terahertz band is the key window connecting light waves and microwaves, and becomes more and more appealing in modern information science [1, 2]. Recent developments on material systems and artificial nanostructure [3], [4], [5], [6] offer intriguing techniques for versatile terahertz applications, ranging from complex signal generation [7], high-precision imaging and spectroscopy [8, 9] and wireless communication [10, 11]. For the state-of-the-arts, integrated nonlinear optical devices in the THz range is one of the most attractive frontiers, driving advances such as ultrashort pulse formation [12], plasmonic emission [13], and electromagnetically induced transparency [14]. However, nonlinear material systems in the terahertz region that allow for integration and are compatible with commercial CMOS technology require further exploration.

Thanks to its bandgap-free Dirac–Fermion distribution, graphene shows exceptionally high nonlinearity in an ultra-broad spectrum, spanning from terahertz to deep ultraviolet [15], [16], [17], [18], [19]. And being an atomically thin nanosheet, graphene is easy to be encapsulated in varied optical structures, and beneficial tremendously to the development of integrated nonlinear optical devices in the terahertz region [20], [21], [22], [23], [24]. However, the ultrathin thickness of graphene also means a limited interaction length, bringing the challenge of weak light–matter interaction, despite its large nonlinear coefficient. A promising strategy has been proposed to overcome this challenge is to combine metastructures with 2D material thus enhancing the light–matter interaction, including nanoparticles [25], hole arrays [26], and grating structure [27]. Benefiting from the excellent manipulation characteristics for electromagnetic waves, meta-structures show great application potential in photonic devices, integrated chip buffers [28], [29], [30], high sensitivity sensors [31, 32] and absorbers. More importantly, there are strong local fields in the sub-wavelength capacitance gap of meta-structure. These tightly bound fields are very sensitive to external excitation and are of great significance for enhancing the light–matter interaction.

Here, we report novel graphene metasurface cavities (GMCs), composed of silicon carbide substrate, 7-layer patterned graphene, and metal micro-ring from bottom to top. The MEMOS-fabricated metal structure enables terahertz field localization, thus enhancing the THz-graphene interactions. By using a co-directionally pumped femtosecond laser beam, we observe remarkable terahertz transparency promotion of terahertz wave (as the probe), due to the ultrafast thermodynamic graphene nonlinearity conductivity. In temporal pumping-probing measurement, such terahertz transparency demonstrates response delay less than 10 ps. Finally, the GMCs also show potential capability for electrical manipulation of terahertz transmission.

## 2 Results

**Figure 1:**
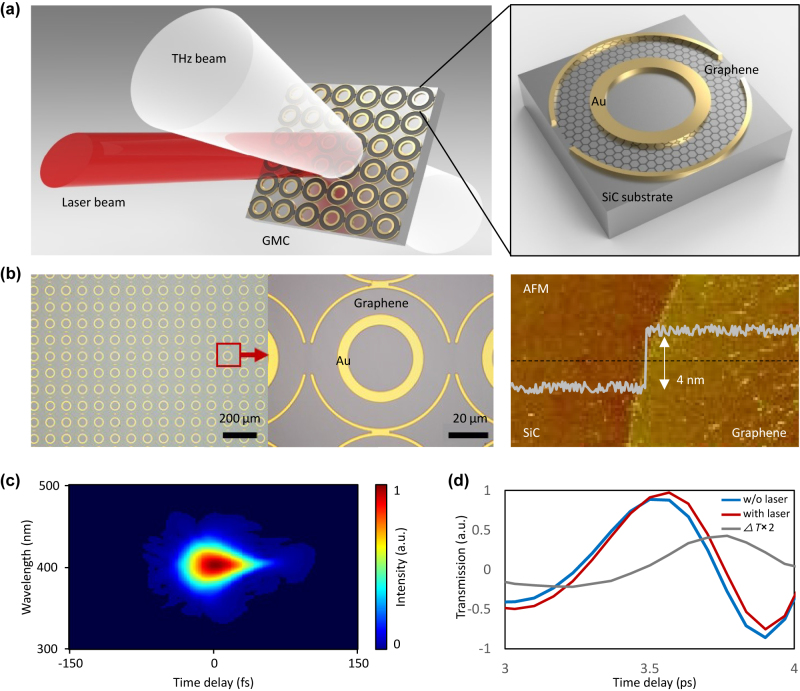
Conceptual design and implementation of the optically controllable terahertz waves. (a) Schematic of the graphene metasurface cavities (GMCs). Circular gold-graphene structures are deposited on a silicon carbide substrate, forming super-interference device for terahertz transmission. (b) Top-view of the GMCs, diameter of gold outer/inner ring is 80/40 μm. The AFM map illustrates the graphene layer number is 7. (c) Measured FROG of the 800 nm titanium-gem mode-locked laser, the pulse width is 35 fs, suggesting peak power up to 10 GW/cm^2^ on a sample. (d) Measured temporal traces of the terahertz transmission through the GMCs on silicon carbide, before (blue) and after laser irradiation (red).

Figure 1a shows our idea of the optically controllable terahertz transmission in the GMCs architecture schematically. 7-layer patterned graphene is grown on a silicon carbide substrate, and a geometrically-designed metallic structure is deposited on it. Each unit cell of the metallic cavity is composed of an inner ring and symmetrically split outer ring, forming a terahertz resonator with strong local field confinement, thus can strengthen the THz-graphene interactions. This structure refers to our previous work, but the inner and outer rings are highly consistent and their parameters are optimized (see Supplementary Section 1) [33]. We use multilayer graphene rather than monolayer graphene, because in the optical penetrating scheme, multilayer graphene ensures higher optical absorption for nonlinear interactions, considering πα is only 2.3% for each graphene layer. An 800 nm (375 THz) pulsed laser pump and a pulsed terahertz wave (probe) are simultaneously focused on the GMCs, with spot-size ≈7 mm^2^. Free carriers in doped graphene absorb photon energy and convert into electron heat via intraband optical conductivity mechanism, which is related to generation of hot electrons, carriers scattering and lattice vibrations [34]. The thermalization of charge carriers dominates the terahertz response, rather than the creation of initial electron–hole pairs, resulting in transient electron distribution that inhibiting graphene’s terahertz conductivity and power absorption. The degradation of graphene conductivity is characterized by the change in Drude conductivity [34, 35]:

(1) $$\Delta \sigma \left(\omega \right)=\frac{D\tau }{1-i\omega \tau }+\frac{iF\omega }{\left(\omega^{2}-\omega_{0}^{2}\right)+i\omega \gamma }$$

where *D* is Drude weight accounting for the dynamic phenomena, and the second term is the Lorentz term (see Supplementary Section 1). This nonlinear effect enables intensity enhancement of the terahertz wave, $\Delta T\propto M\left|\Delta \sigma \right|I_{P}I_{\mathrm{THz}}$ , here Δ*σ* is effective degraded conductivity of the GMCs, *I*_*p*_ and *I*_*THz*_ is the original intensity of the pump and terahertz wave respectively, and *M* is the loss coefficient. In this process, the coupling effect between the two rings provides a considerable local field and promotes the THz-graphene interaction. Specifically, utilization of the metasurface cavity structure helps to increase the *I*_*THz*_ while suppressing the linear loss, offering higher enhancement efficiency. Detailed theoretical analysis is shown in Supplementary Section 1.

Figure 1b presents the top-view optical micrograph of nanofabricated graphene metasurface cavities. On the silicon carbide substrate, the rings are neatly arrayed, where the bright yellow rings are metallic patterns as shown in the zoomed picture. The concentric ring pairs are fabricated by gold, where the outer one is a symmetrical splitting ring with a gap of 8 μm. The diameter of the inner/outer ring is 40 μm/80 μm, with a width 6 μm/2 μm, respectively. The multilayer graphene is located between the inner ring and the outer ring. In nanofabrication, a large-scale graphene multilayer (1 × 1 cm^2^) is grown on the 4H-SiC substrate by chemical vapor deposition method directly [36, 37]. The AFM map illustrates the film thickness ≈4 nm, suggesting the graphene layer number is 7, and further verified by our X-ray photoelectron spectroscopy in Supplementary Section 2. For measuring the induced terahertz transparency, we use a mode-locked femtosecond pump laser with a repetition rate 1 kHz, and a terahertz pulse signal generated by the pump laser. Both of them are launched vertically onto the GMCs. The detailed experimental setup is shown in Supplementary Section 3. Figure 1c maps the retrieved FROG profile of the pump laser; the temporal pulse duration is 35 fs. The maximum peak power of the pump laser reaches 10 GW/cm^2^. And the power density is much lower than the threshold of the intrinsic nonlinearity of gold, which could prevent the response of gold to influence the THz response [38]. In Figure 1d, we show the THz wave in the time domain passing through the GMCs sample before and after laser irradiation. After the laser irradiation, the THz transmission increases, indicating that the multi-layer patterned graphene still well maintained the property of hot electron energy transfer. The grey line shows the difference of THz temporal traces before and after laser irradiation (*∆T = T*_red_ *− T*_blue_, multiplied by two for clarity).

**Figure 2:**
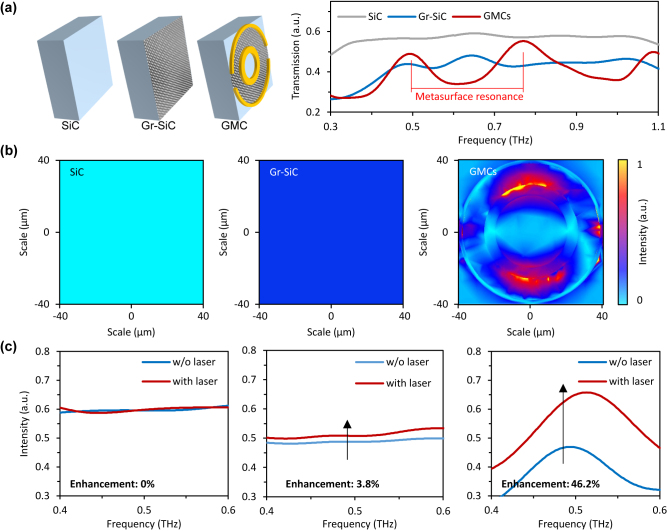
Observation of the dramatic terahertz boost. (a) Via designing the geometry of the metasurface superstructure, we enable terahertz resonance, with *Q* factor ≈ 10. Under strong excitation, it gathers higher energy at 0.5, 0.8, and 1.1 THz. (b) Simulated electrical field distributions of a terahertz wave (0.5 THz). The field intensity in the GMCs is much stronger than in ordinary SiC or Gr-SiC, providing conditions for optical nonlinear regulation. Color bar: Normalized THz intensity. (c) Measured optical tuning. After 300 mW laser irradiation, terahertz transmission of the GMCs increases from 0.45 au–0.658 au, approaching 46.2% enhancement at the frequency 0.5 THz. Here the minor resonance shift is induced by thermal effect.

In Figure 2, we demonstrate the performance of the THz enhancement, by comparing the terahertz wave transmissions in the bare SiC substrate, the graphene deposited SiC (Gr-SiC), and the GMCs (Figure 2a). First, their intrinsic transmissions are measured. For the broadband terahertz signal covering 0.3–1.1 THz, its transmitting spectrum is flat, with intensity 0.55 au. Passing through the GrC, the detected transmission is 0.42 au on average, due to the absorption loss in graphene. In the GMCs, due to the carefully designed meta geometry, we realize observable resonant peaks at 0.5 THz, 0.8 THz, and 1.1 THz, with a quality factor of about *Q* = 10. The meta-cavity induced resonance enhances terahertz wave intensity at the selected frequencies, thus helping to promote the nonlinear THz-graphene interaction.

Figure 2b presents the simulated electrical field distributions of a terahertz wave with 0.5 THz frequency in the three structures. Compared with the bare SiC substrate and the Gr-SiC, The GMCs gathers high local field energy at the metal structure. Specifically, the terahertz wave excites the dipole resonant modes of rings, and a resonance peak is induced through the mutual coupling effect, with a strong local field bounded in the gap. In this case, the substrate acts as a resonant cavity, which contributes to the metasurface to collect local field energy. Figure 2c plots the measured optical tuning, with a 300 mW laser irradiation. In the bare SiC, the terahertz transmittance remains constant when the pump laser is on. In the Gr-SiC, driving by the laser pump, the transmittance of the wave at 0.5 THz increases by 3.8% (from 0.48 au–0.507 au), due to the thermalized electron gas of graphene. While in the GMCs, thanks to the local field resonance, the nonlinear interaction is enhanced, leading to the terahertz transmission further increment, from 0.45 au–0.658 au, the boosting ratio reaches 46.2%.

To further understand the terahertz transparency, we investigated the saturable absorption of GMCs by monitoring both the pump power and the terahertz power dynamically *in-situ*. For graphene with intrinsic doping rate *E*_*F*_ < 1/2*hf*_*p*_, when increasing pumping power, the saturable absorption is caused by the Pauli blocking of inter-band transitions [39, 40], and usually has a smaller threshold. With the on-sample pump power increasing, the graphene conductivity decreases due to the hot electron generation, thus the absorption of both the pump laser and the terahertz wave reduces. In this process, one can observe the transmission of both the pump and the terahertz wave boosting.

**Figure 3:**
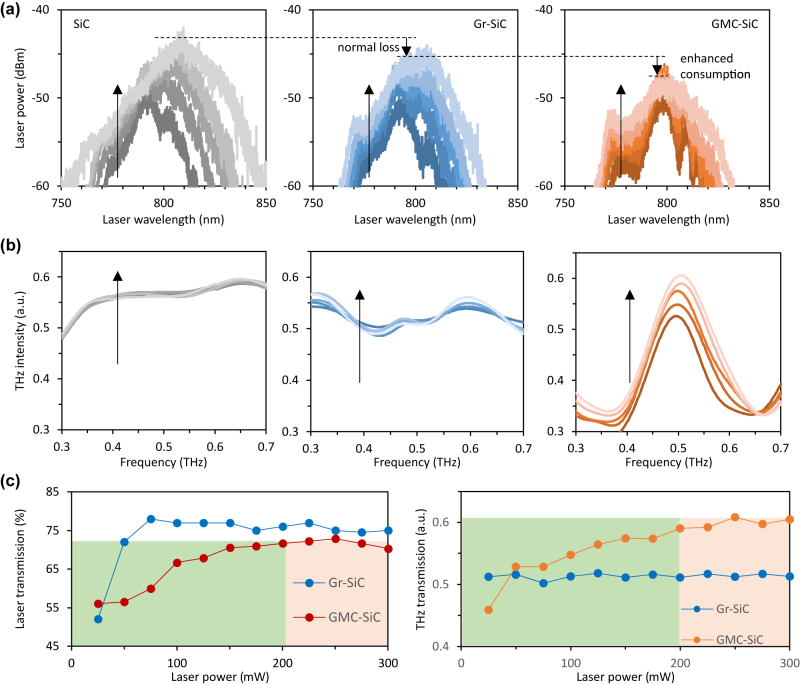
Nonlinearity transmission of SiC, Gr-SiC, and GMCs. (a) Detected laser transmission spectra of the SiC, the Gr-SiC, and the GMCs, all interacting with fixed input terahertz intensity (0.75 au). (b) Detected terahertz wave transmission spectra of the SiC, the Gr-SiC, and the GMCs, all interacting with fixed input terahertz intensity (0.75 au). In (a) and (b), black arrows demonstrate we rise the laser power from 50 mW to 250 mW, with gradient 50 mW per curve. (c) Summarized correlations of ‘laser power versus laser transmission’ and ‘laser power versus terahertz transmission (@0.5 THz)’. From 25 to 300 mW, graphene’s saturable absorption contributes to terahertz transmission enhancement in both the Gr-SiC and the GMCs. As reaching the saturable absorption threshold, GMCs owns higher pump consumption and terahertz enhancement than Gr-SiC.

We demonstrate the transmission spectra of both the pump laser and the terahertz wave in Figure 3a and b, during the pump power increment process. From left to right, the panels plot the cases in the SiC, the Gr-SiC, and the GMCs, respectively. In the measurement, the input terahertz intensity is fixed, while the average pump laser power is tuned from 50 mW to 250 mW. In Figure 3a, an increasing pump input power corresponds to an increasing transmission pump power linearly in the SiC, with a fixed transmittance. Relatively, in the Gr-SiC, the detected transmitting pump power is lower; this is majorly due to the graphene additional absorption. In the GMCs, when the pump laser power increases from 200 mW to 250 mW, the peak of the detected pump transmission in the GMCs fell by 1 dBm, due to the GMCs-enhanced laser consumption. Accordingly, we show the measured terahertz wave transmissions in Figure 3b. In the bare SiC, with increasing laser power, the terahertz transmission keeps no change. In the Gr-SiC, the promotion of laser power brings a slight increment of terahertz transmission (for instance at 0.5 THz, the detected intensity moves from 0.511 au–0.516 au when the laser power goes from 50 mW to 250 mW), majorly due to reduction of the graphene terahertz conductivity. This effect is more pronounced in the GMCs where the larger change in terahertz transmission under a high laser power is obvious: with the pump laser power increasing from 50 mW to 250 mW, transmitting intensity of the terahertz wave at 0.5 THz frequency boosts from 0.52 au–0.65 au and then the detected terahertz transmission keeps on 0.65 au level. It should be noted that the active graphene area is 1−*η* = 49% in the GMCs sample, but its tunability range is over 20 folds larger than the case in the Gr-SiC. Such a remarkable enhancement is caused by the field-enhanced light–matter interaction.

More intuitively, we summarize the correlation of the laser power and the optical transmissions in Figure 3c. The left panel shows the laser transmission (%) while the right panel shows the terahertz transmission (au) at the frequency of 0.5 THz. When the laser power promotes from 0 to 75 mW, we observe the laser transmission increase in both the Gr-SiC. But in the GMCs, due to the meta-cavity-resonance induced high-density of local field intensity, we can see obvious higher laser consumption and sustainable increment of the terahertz transmission. When the laser power is higher than 200 mW, the graphene absorption has been already saturated; hence the laser transmission becomes a flat curve while the enhancement of the terahertz transmission no longer lasts.

## 3 Discussion

**Figure 4:**
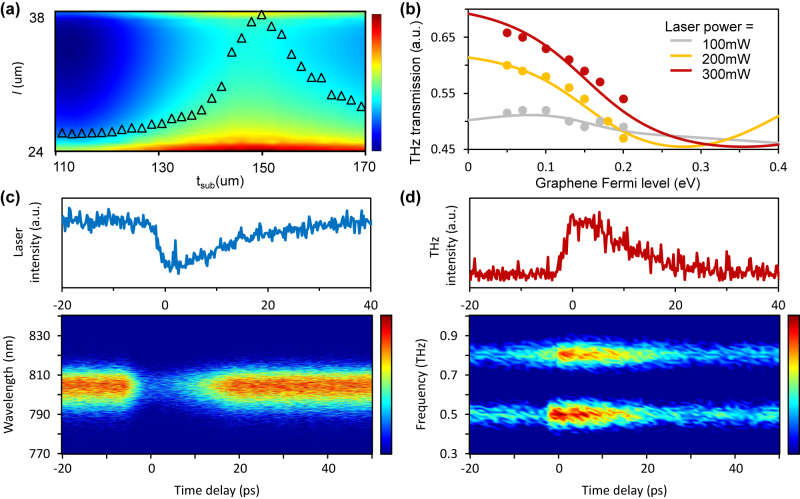
Performance of the optical tuning for terahertz wave. (a) Field confinement in the gap between the rings plotted along the gap (vertical axis) for different t_sub_ at 0.5 THz. Symbols show the integration of the nonlinear field along *l* versus substrate thickness (t_sub_). (b) Measured (symbols) and calculated (lines) terahertz transmission, in the GMCs samples with varied graphene Fermi levels. (c) and (d) Ultrafast response of the laser-terahertz interaction, measured by the pumping-probing scheme, under 300 mW laser power. It further verifies the terahertz amplification is derived from laser consumption. The total nonlinear relaxation delay is <10 ps, suggesting its potential for THz control with tens of GHz bandwidth. Color bar: normalized intensity.

For better understanding the enhancement of the metasurface metal cavities, we perform simulations; the results are shown in Figure 4a. The relationship between the field enhancement caused by the GMCs and the graphene is given by [41]:

(2) $$E=\underset{l}{\int }\varepsilon \left(\omega \right)\eta_{\omega }\left(x\right)E_{0}dx$$

where *l* is the length of simulation region used for integration between the inner and outer rings along the direction defining polarization electric field. $\varepsilon \left(\omega \right)$ is the effective permittivity and $\eta_{\omega }\left(x\right)$ is a dimensionless quantity that represents the enhancement in the electric field amplitude that acts on the graphene layer relative to the incident field amplitude, which is obtained by using a commercial software CST Microwave Studio. The GMCs are regarded as effective optical resonant cavities with boundary conditions determined by the metasurface: when the phase difference is $Re\left(k\left(\omega \right)\right)\cdot t_{\text{sub}}=n\pi$ (*n* is integer), corresponding to resonance condition, the field energy will be captured by the metal structure, via momentum matching. The simulated results in Figure 4a show that the enhancement is mediated by the metasurface cavities, which amplify the electric near-field in the graphene region. The light–matter interaction of the system could be greatly enhanced by properly choosing geometric parameters of metasurface cavity. Besides optical tunability via modifying the auxiliary laser power, the tunable terahertz transmission is also obtainable via changing the graphene Fermi level electrically. We calibrate the *E*_*F*_ of our graphene samples in GMCs via *in-situ* Raman spectroscopy (see Supplementary Section 2), an estimated momentum scattering time, and picosecond cooling dynamics are used as the input parameters for the graphene simulation. Picking the cases when the laser power is 100 mW, 200 mW, and 300 mW, we measure the tunable performances; results are plotted in Figure 4b. When increasing the graphene Fermi level from 0 eV–0.2 eV: the measured terahertz transmission at 0.5 THz decreases from 0.66 au–0.54 au with 300 mW laser power.

Such terahertz transparency boosting also demonstrates a unique fast response. By using a controllable free-space delay line, we show the temporal delay of the terahertz enhancement and recovery in our pumping-probing measurement (Figure 4c and d). Experimental details are shown in Supplementary Section 3. The blue trace and the red trace plot the temporal evolution of the transmitting laser intensity and the transmitting terahertz wave intensity correspondingly. Once the laser pulse and the terahertz pulse overlaps (at the time offset 0 ps), the electron gas of graphene consumes the pumping power, meanwhile enhancing the terahertz wave. When the dynamically delayed laser pulse moves across the terahertz pulse, we detect a dip in the optical band and a peak in the terahertz band. As aforementioned, the pulse duration of the pump laser is 35 fs while the pulse duration of the terahertz wave is ≈1.2 ps with an asymmetric tail (Figure S6), the dual-pulse cross-correlation would last for a few picoseconds. In the pumping-probing measurement, the temporal width dip/peak is <10 ps, suggesting that the switching is <8 ps. The measured Δ*I*_*THz*_ and Δ*I*_*p*_ traces are asymmetric temporally, meeting the pulse shape of the terahertz wave. The contour maps present the evolution of time-frequency transmission amplitude with different pump-probe time delay over the whole scanning cycle, in the time-offset region −20–40 ps, the laser pulse gradually approaches the terahertz pulse, and then moves away, during this process both the laser spectrum and the terahertz resonance in the GMCs keeps well. In Figure 4d, the meta-cavity resonances at 0.5 THz and 0.8 THz are clear. Such a single picosecond level response suggests a powerful way that one can use a fast-optical source to control the terahertz transmission dynamically, with unprecedented speed over 100 GHz, only limited by the relaxation delay of the graphene carriers. Related to conventional terahertz tuning schemes based on thermal or linear-absorption control [42, 43], our technique offers not only broad tuning bandwidth but also a large extinction ratio.

## 4 Conclusions

In this study, we report a graphene meta-cavity device for ultrafast terahertz transparency boosting. Leveraging the nonlinearity conductivity in graphene and the meta-cavity based field convergence, we realize up to 46.2% enhancement of terahertz transmission. Such result demonstrates uniquely tunable capabilities such as ultrafast optical on–off operation with delay <10 ps, and electro-optical modulation potential via engineering the graphene Fermi level. This combination of two-dimensional meta-surface and ultrafast nonlinear photonics bridges the light wave and terahertz wave in nanoscale, develops our understanding of graphene optoelectronics in THz region, and offers new schemes for ultrafast terahertz control, may open a novel platform for future advanced technologies ranging from ultrahigh-speed terahertz communication and signal processing.

## 5 Method

### 5.1 Nano-fabrication of the graphene meta-cavities on silicon carbide

The GMCs used in this work is fabricated on quasi-free-standing multilayer graphene by epitaxial growth on Si-terminated 4H-SiC (001) substrate. This method can grow a rare large-area graphene layer of 1 × 1 cm^2^. The gold film is pre-deposited on the graphene as protection to keep the graphene away from any possible contamination. After that, the graphene together with the protective layer is patterned by photolithography and oxygen plasma etching. Through photoetching, electron-beam evaporation, and lift-off processes, a complex metal layer of Au/Ti (200/10 nm) is orderly deposited onto the sample to form a metal structure. The improved fabrication approach is an easy operation. Most importantly, it keeps the graphene layer away from the undesired photo-resist contamination, surface air oxidation and damage during the fabrication process. The fabrication process for the GMCs and characterization of the nanofabrication is shown in Supplementary Section 2.

### 5.2 Experimental set-ups and measurement details

A Ti: sapphire laser (Newport Corporation, SOL-ACE35F1) with central wavelength 800 nm, 1 KHz repetition rate and 35 fs pulse width is divided into two paths. The reflected beam is focused on the ZnTe crystal through a chopper, and a broadband terahertz wave of 0.2–3 THz is generated due to the optical rectification effect. A silicon wafer is placed behind the first parabolic mirror to block the 800 nm red light, and only allow the terahertz wave to pass through and reach the sample surface. The temporal delay between THz pulses and laser pulses is tuned with the optical delay line (Newport M−436). When the two pulses overlap in time, and the phase matching condition is satisfied, the SA could occur. The variable optical attenuator behind the optical delay line is used to adjust the optical power on the sample, and the optical signal after passing through the sample is detected by the Optical Spectrum Analyzer (OSA, Yokogawa AQ6370D) through the fiber collector.

## Supplementary Material

Supplementary Material Details
