# Supplementary material for: Ultrafast terahertz transparency boosting in graphene meta-cavities
Source: Nanophotonics. 2022 Nov 16;11(21):4899–907. doi: 10.1515/nanoph-2022-0511 (PMC11614338; doi:10.1515/nanoph-2022-0511)
Supplement: Supplementary file 1 — Supplementary Material Details [file j_nanoph-2022-0511_suppl_001.pdf]

Supplementary Information for

Ultrafast terahertz transparency boosting in graphene meta-cavities

Lan Wang[1,2#], Ning An[3#], Sen Gong[2], Xuan Sheng[2], Yiwei Li[3], Baicheng Yao[3], CuiYu[4*], Zezhao He[4], Qingbing Liu[4], Zhihong Feng[4], Taiichi Otsuji[5], Yaxin Zhang[2]**

[1]Yangtze Delta Region Institute (Huzhou), University of Electronic Science and Technology of China, Huzhou, China.

[2]Sichuan Terahertz Communication Technology Engineering Research Center, University of Electronic Science and Technology of China, Chengdu, China

[3]Key Laboratory of Optical Fiber Sensing and Communications (Education Ministry of China), University of Electronic Science and Technology of China, Chengdu, China

[4]National Key Laboratory of Application Specific Integrated Circuit, Hebei Semiconductor Research Institute, Shijiazhuang, China

[5]Research Institute of Electrical Communication, Tohoku University, Sendai, Japan

According to the study of ultrafast transient response revealed by previous work, monolayer and multilayer graphene shows a transient decrease or increase in terahertz conductivity by ultrafast optical excitation [1],[2]. Although a variety of studies have been performed on graphene, very few fundamental studies have been done to explore the possibilities of patterned graphene-based devices for ultrafast photonics [3-7]. Here, we exploit the photoexcitation properties of the graphene to actively modulate the amplitude in a patterned graphene-embedded hybrid device on an ultrafast time scale.

S1 Theoretical analysis and Simulation

Fig. S1a shows a simulation results of the metal structure. Terahertz wave excites the dipole resonant modes of rings, and a resonance peak is induced through the mutual coupling effect, with a strong local field bounded in the gap. In this case, a small change in the graphene conductivity is not sufficient to cause large frequency shifts and mainly induces significant amplitude modulation under optical pumping.

Excluding the effects of graphene, **Fig. S1b** and **c** also shows that the field strength in the gap depends on the width of the rings. The nonlinear response of the system could be greatly enhanced by properly choosing geometric parameters of metasurface cavity.

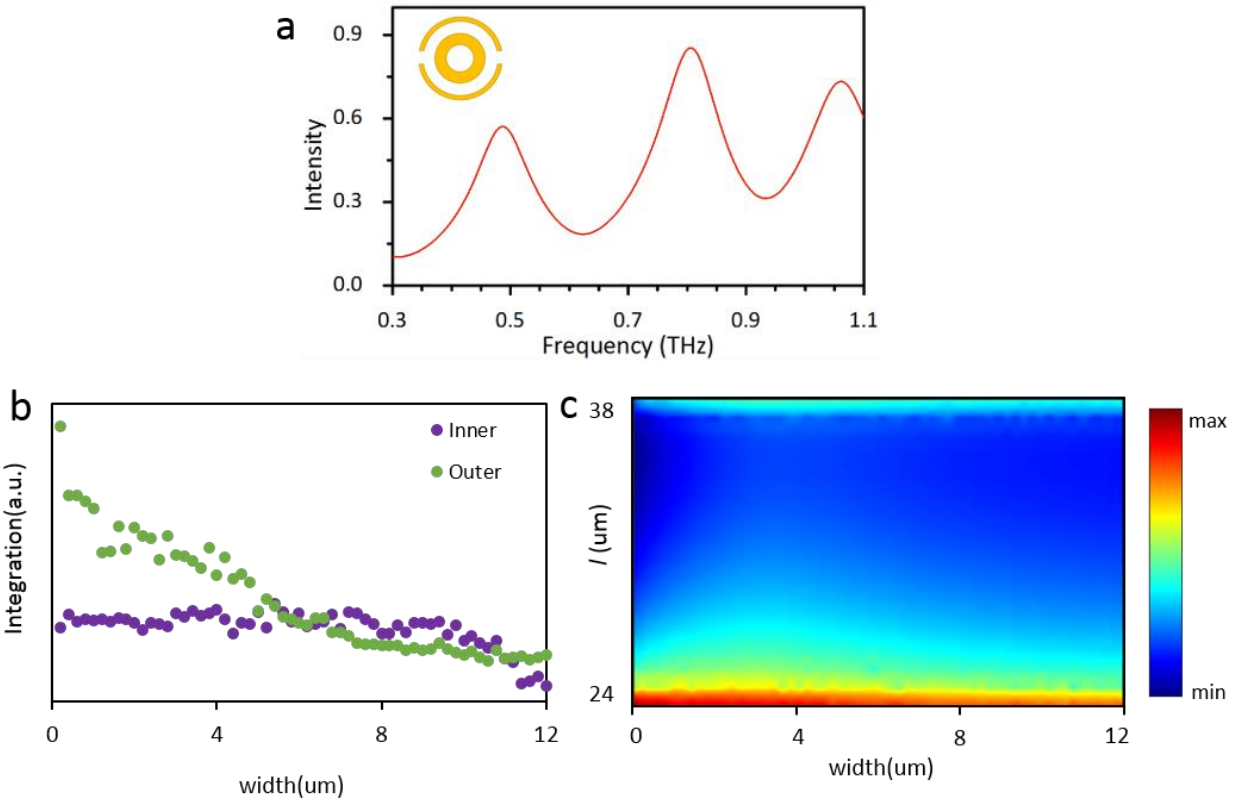

Figure S1. Simulation of SRRs-SiC and terahertz oscillation field enhancement for GMCs. a. The simulated spectra of SRRs-SiC. **b.** The integration of the nonlinear field along *l* versus rings width (*w*). **c.** Field confinement in the gap between the rings plotted along the gap (vertical axis) for different rings width at 0.5 THz.

We calculate the relationship of the Drude weight *D*, the frequency and the decrease in the the real part of graphene conductivity, as shown in **Fig.S2a**. **Fig. S2b** demonstrate the simulated transmission of GMCs. We decrease the graphene conductivity from $3.22G_0$ to $2.38G_0$, corresponding transmission of terahertz waves at 0.5 THz boots from 0.54 a.u. to 0.65 a.u. level.

To explore the underlying physics of the active transparency boosting behavior, the widely used two-oscillator model is adapted to analyze the near-field interaction between the two rings in the unit cell [8],[9]:

$$\begin{aligned} \ddot{x}_1 + \gamma_1 \dot{x}_1 + \omega_0^2 x_1 + \kappa x_2 &= g_1 E, \\ \ddot{x}_2 + \gamma_2 \dot{x}_2 + \omega_0^2 x_2 + \kappa x_1 &= g_2 E \end{aligned} \qquad \text{(S1)}$$

Here x and γ are the amplitude and the damping rate, subscripts 1 and 2 represent the inner and outer resonators, respectively. g is a geometric parameter indicating how strong the resonator couples with the incident electromagnetic wave E. In this equation, the κ denotes the coupling coefficient. Compared with the terahertz wavelength, the thickness of the meta-graphene layer is negligible and the transmission coefficient can be expressed as:

$$t(\omega) = \left| \frac{c(1+n_{sap})}{c(1+n_{sap}) - i\omega\chi_e} \right| \quad \text{(S2)}$$

where n_{sap} is the refractive index of the substrate and c is the light velocity in vacuum. The magnetic susceptibility χ_e is expressed by γ, κ and g, which characterizes the polarization intensity of the metasurface unit cell to the incident electromagnetic field. **Fig.S2c** shows the fitting results to transmission spectra via various graphene conductivity, exhibiting good agreement with the experiment. The fitting parameters γ_1, γ_2 and κ are shown in **Fig.S2b**. It was observed that the damping coefficients γ_1 and γ_2 do not significantly change with the decrease of graphene conductivity, while the coupling coefficient κ increases significantly and then remains almost constant. Therefore, utilization of the metasurface cavity structure helps to increase the terahertz intensity via coupling effect, offering higher enhancement efficiency.

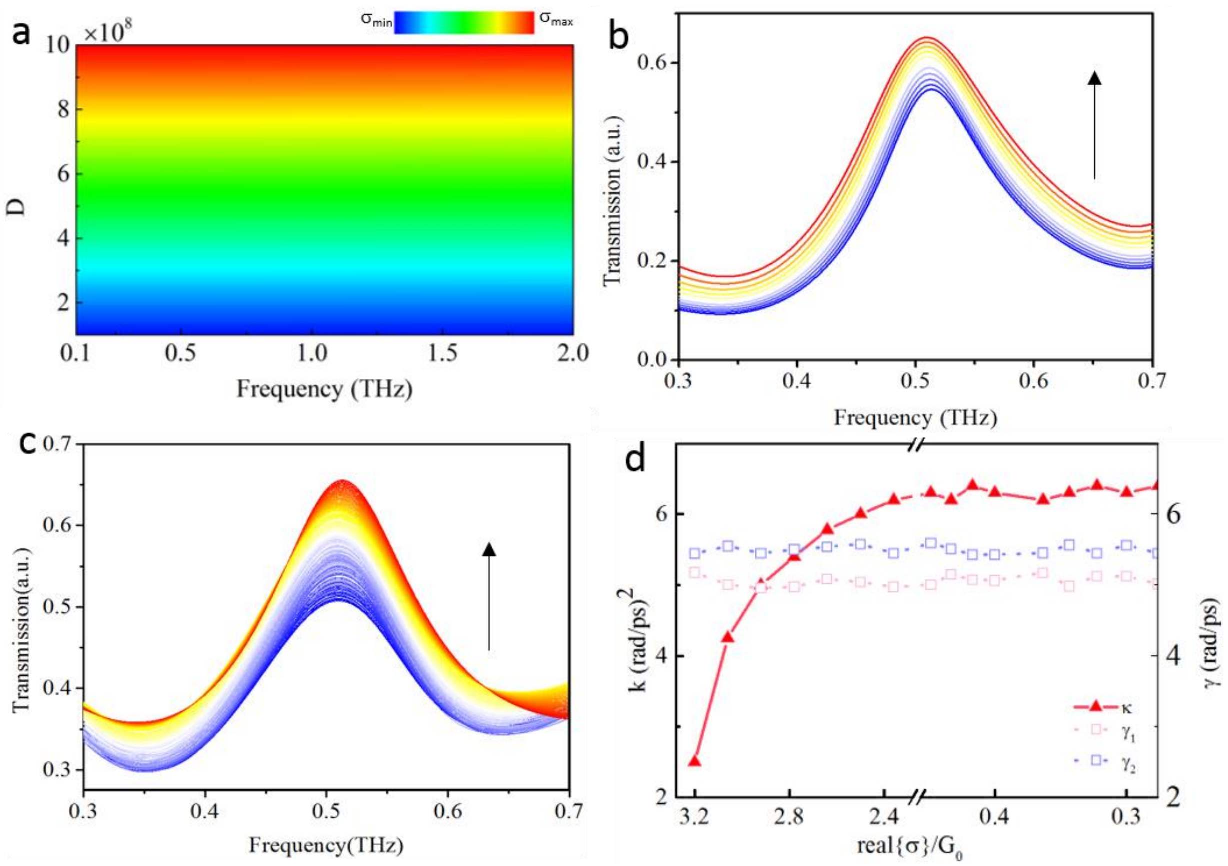

Figure S2. **a**, The relationship of the Drude weight D, the frequency and the decrease in the real part of graphene conductivity, wherein the oscillation intensity F=4.3×10$^8\Omega^{-1}$S^{-1}; line width γ=11.5 THz; resonant frequency $\omega_0/2\pi$ =2.7 THz. **b**, Simulated transmission of GMCs-SiC. **c** and **d** are fitting transmission coefficients and corresponding parameters under various graphene conductivity, respectively.

S2. Nano-fabrication and characterizations of the graphene metasurface cavities

We fabricate an array of hybrid structure consisting of multilayer graphene and metal resonators by standard photolithography, as shown in **Fig. S3**. First, a large-scale graphene multilayer (1×1 cm^2) is grown on a 4H-SiC substrate by CVD method. Then, a 20 nm-thick Au layer is deposited on the surface of the graphene to prevent it from being contaminated and damaged by PMMA. The PMMA pattern of the graphene region is produced by standard lithography process, and then the Au layer and the graphene layer outside the region are etched using KI+ I_2 solution and oxygen plasma equipment, respectively. Through photoetching, electron-beam evaporation, and lift-off processes, a complex metal layer of Au/Ti (200/10 nm) is orderly deposited onto the sample to form metal structure. The improved fabrication approach is easy operation. Most importantly, it keeps the graphene layer away from the undesired photo-resist contamination, surface air oxidation and damage during the fabrication process.

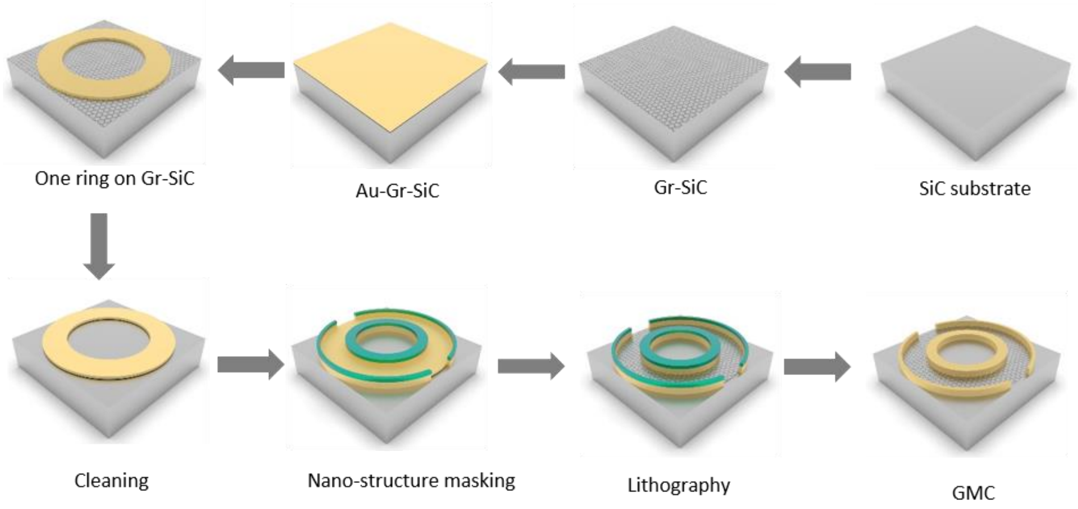

Figure S3. Fabrication. Fabrication flow of the graphene metasurface cavities. Here we show 8 steps to realize the GMCs, by depositing graphene, nano-patterning and lithography etching.

We use X-ray photoelectron spectroscopy to estimate the number of multilayer graphene layers. **Fig.S4a** shows the XPS spectrum of C 1s, as shown by the black line. The spectral line presents two peaks, the first one corresponding to the C in the substrate SiC (282.5eV), and the second peak derived from the sp^2 hybrid C atom in graphene (284.5eV) [10],[11]. Using attenuation models, graphene thickness can be estimated [12]:

$$t_{graphene} = \lambda_C \ln\left(\frac{I^C_{graphene}}{I^C_{SiC} + I^C_{buffer}} + 1 \right) \tag{S3}$$

where λ_c=1.8 is the electron escape depth, $I^C_{graphene}$, I^C_{SiC} and I^C_{buffer} is the integral area of graphene, SiC and C atom in the buffer layer obtained by Gaussian peak division method. The final estimate is about seven layers of graphene. Besides, Raman spectroscopy is measured using Renishaw inVia Raman Microscopes with 532 nm laser excitation at room temperature 300K (**Fig. S4b**). As the Raman spectrum suggests, the dashed orange curve plots the measured result of the SiC substrate, background noise in the band 1500 cm^{-1} to 1900 cm^{-1} is induced by the phonons in SiC. The solid blue curve plots the case of the GMCs, here two dominant Raman peaks of graphene, the G peak (~1582cm^{-1}) and the 2D peak (2696cm^{-1}) are clear, verifying the existence of graphene layers. Furthermore, we measure the Raman spectra of different samples with diverse doping states, as shown in **Fig. S4c** and **S4d**. With varied E_F, spectral shift and intensity alteration of the G peak and the 2D peak in samples are characterized. Typically, for the amplitude of graphene Fermi level $|E_F|$ increases from 0 eV to higher, one can see the intensity increment and spectral right shift on G peak, meanwhile intensity decrement and spectral left shift on 2D peak [13].

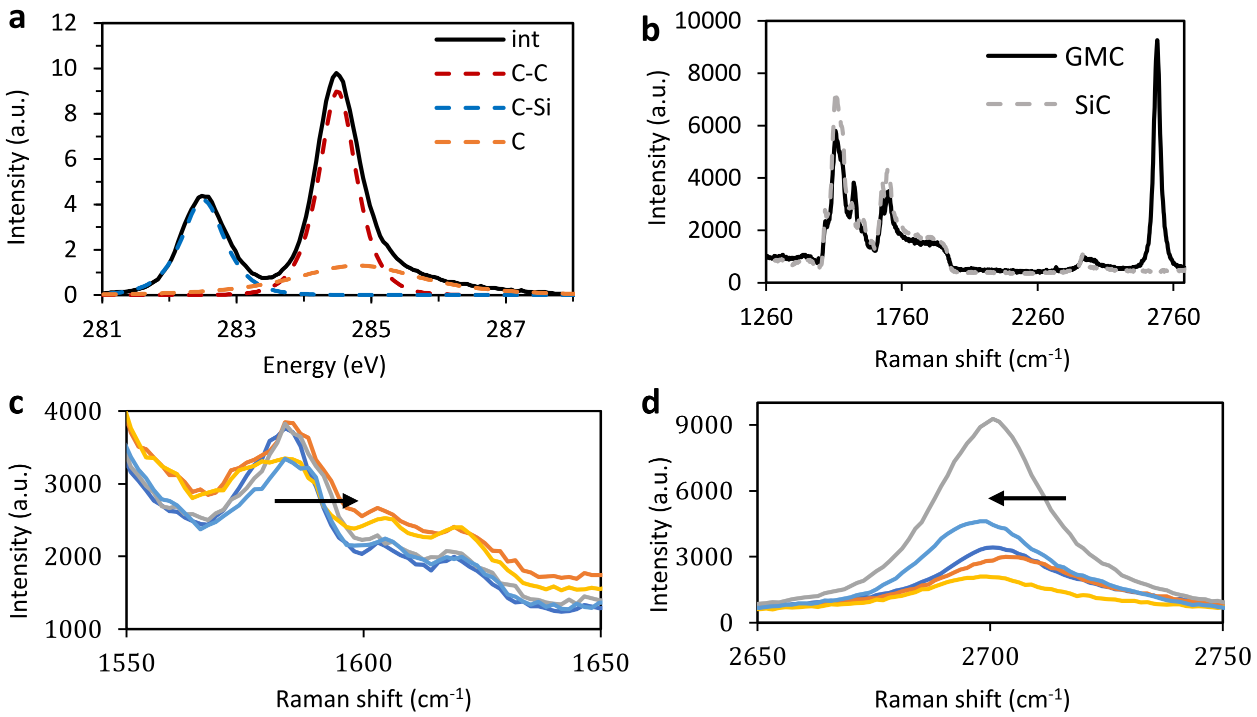

Figure S4. Characterization of the graphene in GMCs. a, The X-ray photoelectron spectroscopy of multilayer graphene. Here the peak at 282.5 eV denotes the C-Si key on boundary, while the peak at 284.5 eV presents the C-C key in graphene. **b**. Raman spectrum of the graphene on the metasurface cavities (solid blue curve) and SiC substrate (dashed orange curve). **c** and **d**. Measured Raman spectra at points with different doping state (or E_F) on different GMCs samples. In c and d, the arrows mark Fermi level increases from 0 to 0.3 eV.

S3. Extended information of experiment

Fig. S5 illustrates the experimental setup. The laser source passes through a beam splitter with the average power of 1kW. 70% of the output of the laser is used for the generation of THz waves. the other part of the laser is used as the pumping light in our GMCs sample. The on-sample spot diameter is 1cm (larger than the terahertz spot diameter of 2~3mm), which ensures the uniform optical excitation on the planar surface of the sample. So, the maximum peak power of the pump laser reaches 10 GW/cm^2 on sample. A silicon wafer (1mm) is placed behind the first parabolic mirror to block the laser, and only allow the terahertz wave to pass through. The temporal delay between THz pulses and laser pulses is tuned with the optical delay line (Newport M-436). When the two pulses overlap in time, the terahertz resonance gradually increased, and the resonance intensity reached the maximum at 0 ps. And we can obtain the obvious THz amplitude enhancement in the spectral domain after using the standard Fourier transform of the terahertz time-domain waveform.

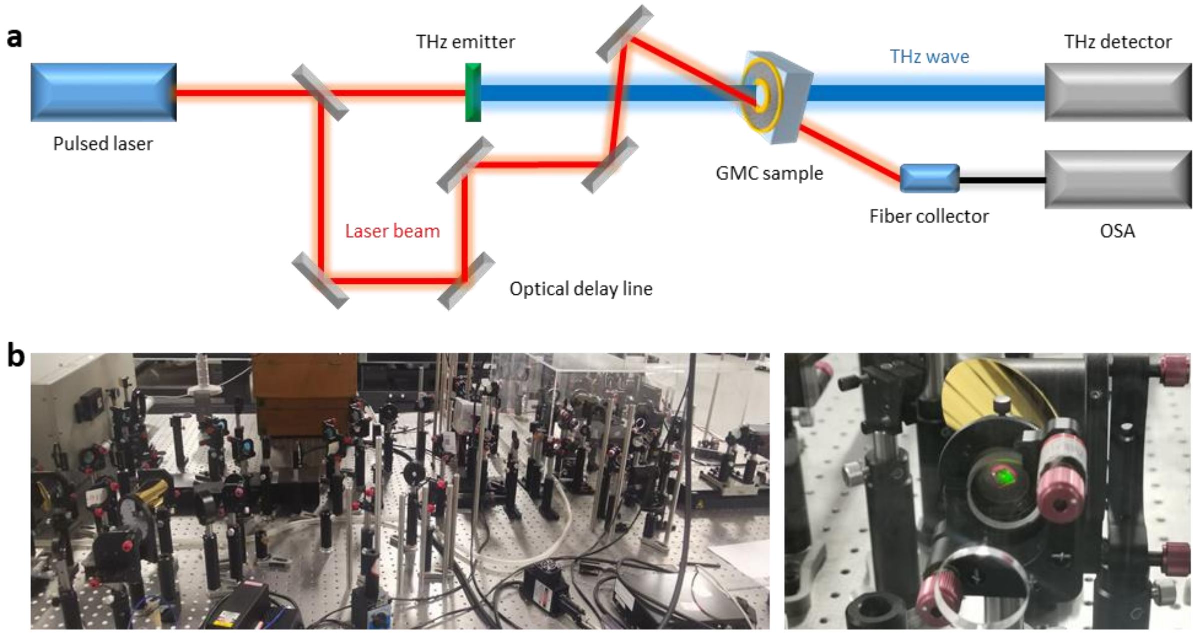

Figure S5. Experimental setup. a. Schematic diagram of the setup, with both terahertz wave excitation and detection. **b**. Pictures of the setup. Left: the whole setup on optical table; right: the GMCs sample on holder.

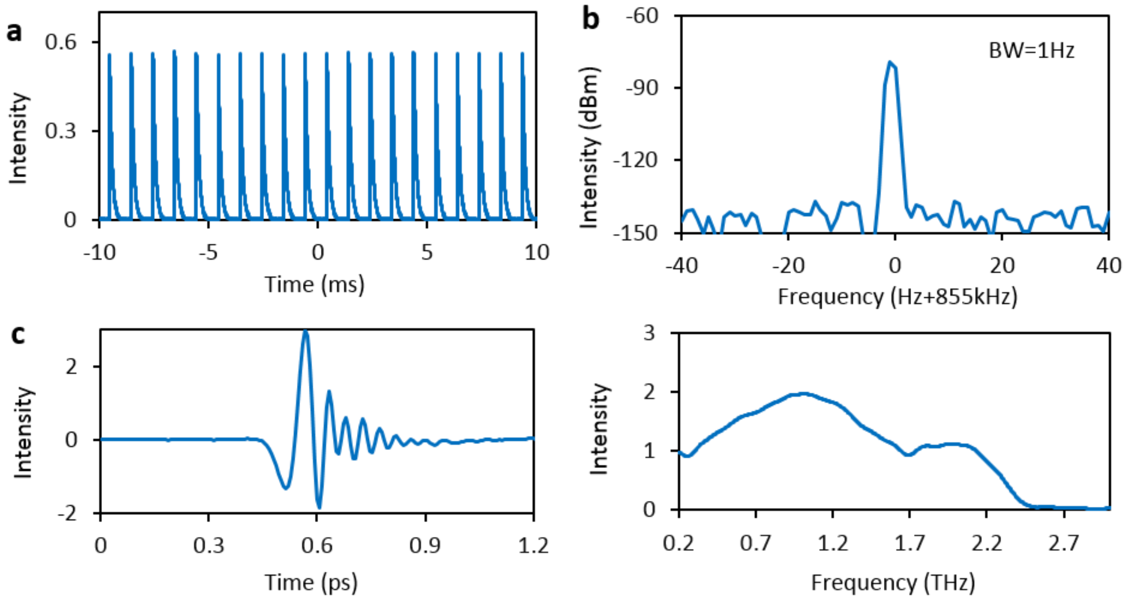

Figure S6. Characterization of the laser source and THz transmission. a. Measured pulses of laser source with a repetition rate of 1kHz. **b.** Measured self-beat-note of the laser source, The SNR is > 60 dB, demonstrating the high stability of the laser source. **c.** Measured temporal traces of the terahertz transmission and the corresponding Fourier transform spectrum.

Here we introduce the light source in detail. A Ti: sapphire laser (Newport Corporation, SOL-ACE35F1) with central wavelength 800 nm and 35 fs pulse duration is used as the laser pump. The time-domain of the laser source is shown in **Fig. S6a**, it illustrates stable pulses of the laser source with a fixed repetition rate of 1kHz. **Fig. S6b** plots the electrical self-beating notes of the laser, the SNR of the beating-line is higher than 60 dB. The laser focuses into a 1 mm thick <110> ZnTe crystal, thus THz wave is generated though optical rectification in ZnTe. In the spectral domain, the generated THz pulses is broadband, from about 200 GHz to typically 3 THz (**Fig. S6c**) [14].

In **Fig. S7a**, we test the long-term power stability of the laser source. The laser power uncertainty is < 0.1 dB with a continuous measurement of 60 minutes at room temperature. Besides, we also measure the stability of the THz intensity generated by the laser through ZnTe (**Fig. S7b**). Due to the fluctuations of environmental temperature and humidity, the uncertainty of the THz signal is a bit higher than the laser source (~0.25dB).

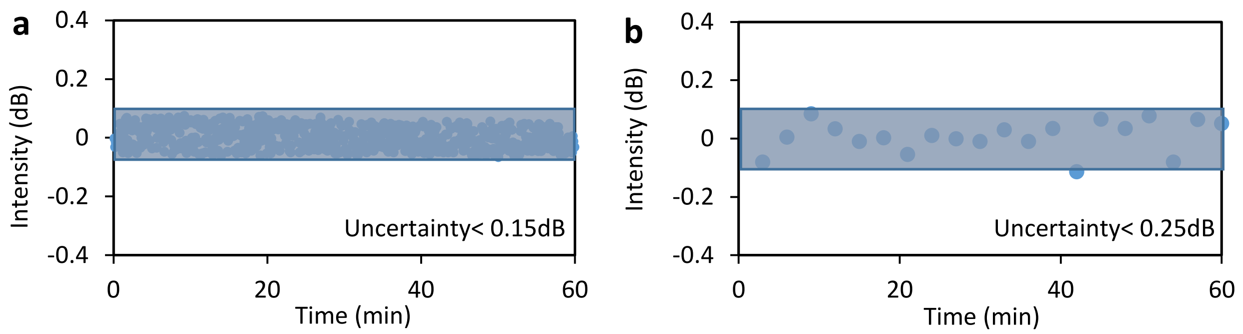

Figure S7. Long-term stability and reliability of the laser pump and the terahertz source. a. The laser power is measured continuously in 1 hour, and the result shows the power uncertainty <0.15dB. **b**. The THz intensity is measured continuously in 1 hour, and the result shows the power uncertainty <0.25dB

References:

1. Shi, S-F., et al, "Controlling graphene ultrafast hot carrier response from metal-like to semiconductor-like by electrostatic gating," *Nano letters*, vol.14, pp. 1578-1582, 2014.
2. Lu, C., Hu, X., Zhang, F., Yang, H., and Gong, Q, "Multilayer Graphene: Polycrystalline ITO for Ultralow□ Power Active Control of Polarization□Insensitive, Metamaterial□Induced Transparency," *Advanced Optical Materials*, vol. 2, pp. 1141-1148, 2014.
3. Yao, B. et al, "Broadband gate-tunable terahertz plasmons in graphene heterostructures," *Nat. Photonics*, vol. 12, pp. 22–28, 2018.
4. Ren, Q., You, J. W. and Panoiu, N. C, "Large enhancement of the effective second-order nonlinearity in graphene metasurfaces," Phys. Rev. B, vol. 99, pp.1–10, 2019.
5. Yao, X., Tokman, M. and Belyanin, A, "Efficient nonlinear generation of THz plasmons in graphene and topological insulators," *Phys. Rev. Lett*, vol.112, pp. 1–5,2014.
6. Alonso Calafell, I. et al, "Giant enhancement of third-harmonic generation in graphene–metal heterostructures," *Nat. Nanotechnol*, vol. 16, pp. 318–324, 2021.
7. Sergey Kovalev, et al, "Electrical tunability of terahertz nonlinearity in graphene," *Science advances* , vol.7, p. eabf9809, 2021.
8. Gu, J, et al, "Active control of electromagnetically induced transparency analogue in terahertz metamaterials," *Nature communications*, vol. 3, pp. 1-6, 2012.
9. Zhu, Z, et al, "Broadband plasmon induced transparency in terahertz metamaterials," *Nanotechnology*, vol. 24, p. 214003, 2013.
10. Michon, A. et al, "Direct growth of few-layer graphene on 6H-SiC and 3C-SiC/Si via propane chemical vapor deposition," *Appl. Phys. Lett*, vol. 97, pp. 2008–2011.
11. Emtsev, K. V., Speck, F., Seyller, T., Ley, L. and Riley, J. D, "Interaction, growth, and ordering of epitaxial

graphene on SiC{0001} surfaces: A comparative photoelectron spectroscopy study," *Phys. Rev. B* ,vol. 77, pp.1–10. 2008.

12. Hattori, A. N. et al, "Structure and magnetic properties of mono- and bi-layer graphene films on ultraprecision figured 4H-SiC(0001) surfaces," *J. Nanosci. Nanotechnol*, vol. 11, pp. 2897–2902, 2011.

13. An, N. et al, "Electrically Tunable Four-Wave-Mixing in Graphene Heterogeneous Fiber for Individual Gas Molecule Detection," *Nano Lett*, vol. 20, pp. 6473–6480, 2020.

14. Zhao, Z. Y., Hameau, S., Voos, M. and Tignon, J, "THz generation by optical rectification and competition with other nonlinear processes," *Chinese Physics Letters*, vol. 25, p. 1868, 2008.
